# New Radiometric Ages for the BH-1 Hominin from Balanica (Serbia): Implications for Understanding the Role of the Balkans in Middle Pleistocene Human Evolution

**DOI:** 10.1371/journal.pone.0054608

**Published:** 2013-02-06

**Authors:** William J. Rink, Norbert Mercier, Dušan Mihailović, Mike W. Morley, Jeroen W. Thompson, Mirjana Roksandic

**Affiliations:** 1 School of Geography and Earth Sciences, McMaster University, Hamilton, Ontario, Canada; 2 Institut de Recherche sur les Archéomatériaux, Centre de Recherche en Physique Appliquée à l’Archéologie, Maison de l’Archélogie, Pessac, France; 3 Department of Archaeology, Faculty of Philosophy, Belgrade University, Cika Ljubina, Belgrade, Serbia; 4 Human Origins and Palaeo-Environments (HOPE) Research Group, Department of Social Sciences, Oxford Brookes University, Gipsy Lane, Oxford, United Kingdom; 5 Department of Medical Physics and Applied Radiation Sciences, McMaster University, Hamilton, Ontario, Canada; 6 Department of Anthropology, University of Winnipeg, Winnipeg, Manitoba, Canada; Illinois State University, United States of America

## Abstract

Newly obtained ages, based on electron spin resonance combined with uranium series isotopic analysis, and infrared/post-infrared luminescence dating, provide a minimum age that lies between 397 and 525 ka for the hominin mandible BH-1 from Mala Balanica cave, Serbia. This confirms it as the easternmost hominin specimen in Europe dated to the Middle Pleistocene. Inferences drawn from the morphology of the mandible BH-1 place it outside currently observed variation of European *Homo heidelbergensis*. The lack of derived Neandertal traits in BH-1 and its contemporary specimens in Southeast Europe, such as Kocabaş, Vasogliano and Ceprano, coupled with Middle Pleistocene synapomorphies, suggests different evolutionary forces acting in the east of the continent where isolation did not play such an important role during glaciations.

## Introduction

The Middle Pleistocene has become increasingly recognized as an important period in the biocultural evolution of our lineage. Lebel et al. recognize “exaggerated encephalization, the controlled use of fire, temperate zone geographic dispersals, varieties of prepared core lithic reduction techniques, the development of effective (predatory and defensive) weaponry, and regional differentiation of human populations” among relevant developments [1]. In Europe, the Middle Pleistocene is generally associated with *Homo heidelbergensis* [2], a species that was, and continues to be, the subject of substantial controversy regarding its morphology, geographic spread and phylogenetic position (for recent critical overviews see [3], [4]). Although some consider *Homo heidelbergensis* as once extending across the Old World [5], it is more commonly regarded as a European Middle Pleistocene phenomenon, often associated with an early stage in Neandertal evolution [6]. Cartmill and Smith have suggested that the question of *H. heidelbergensis* taxonomy is not easily solved and advise that we should be referring to these specimens as Heidlebergs [7], while Stringer [4] recently concluded that questions relating to the phylogenetic position of this species and its differentiation from *H. rhodesiensis* and other Middle Pleistocene hominins might never be answered since “… these fossils are close to the morphotype expected in the common ancestor of Neanderthals and ‘modern’ *H. sapiens*” [3]. As an encephalized, non-specialized hominin, *H. heidelbergensis* could be ancestral to either or both Neandertals and modern human. However, since all of the European specimens included in the *H. heidelbergensis* hypodigm present some Neandertal traits, it is commonly considered as a chronospecies [8], which over time acquired increasingly more specialized Neandertal morphology [5] in the glacial quasi-isolation of Western Europe. It is increasingly evident that the species level might not be the most productive level of discourse when discussing hominin populations in the Middle Pleistocene [9], [10]. A more appropriate level of comparison relies on the “paleo-deme” or “p-deme” concept [11] that allows us to distinguish between local populations and discuss their possible phyletic relationships without implying (or rejecting) speciation events.

Against this background, every new fossil from the Balkans, where Pleistocene populations were not subject to the same levels of isolation experienced by their western counterparts during glacial periods, could contribute substantially to our understanding of hominin evolution in Europe. A left semi-mandible, BH-1, from Balanica, Serbia [12], is particularly important as it represents the only Middle Pleistocene hominin specimen from the Central Balkans.

The mandible was excavated at Mala Balanica cave, which together with Velika Balanica forms the Balanica Cave Complex, located in Sićevo Gorge, south Serbia (N43°20.211’, E22°05.115’). This cave complex has been the focus of systematic archaeological excavations since 2004 [13]. Middle Paleolithic artifacts were recovered from the upper levels of both caves and a hominin mandible in the lower stratigraphic level of Mala Balanica, 1.5m below the artifact bearing levels. The excavations are ongoing and bedrock has not been reached in either cave. The detailed characteristics of the sedimentary sequence and details of morphology of the BH-1 mandible are described elsewhere [12]. In this paper we present new ages relevant to the age of the mandible that were obtained by ESR-US and ESR-CSUS dating of tooth enamel, ^230^Th/^234^U closed system dating of speleothem carbonate, and infrared/post infrared luminescence dating of cave sediment. We also examine its morphology in the light of an increasing number of Middle Pleistocene hominins in the southeast of the continent.

## Materials and Methods

**Figure 1:**
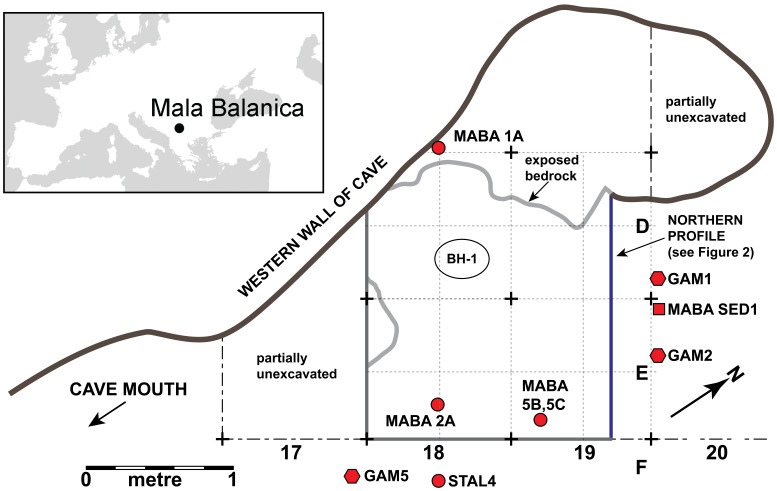
Location of the site and the distribution of samples in the cave. Upper left panel: location of Mala Balanica in southwestern Europe. Right panel: plan of Mala Balanica indicating excavated areas superimposed on the excavation grid square identifiers (D, E, F, 17, 18, 19), and locations of dated teeth, sediment, and speleothem samples, and in-situ gamma measurement locations (GAM). Note position of northern profile here (in blue), which is depicted in Figure 2.

**Figure 2:**
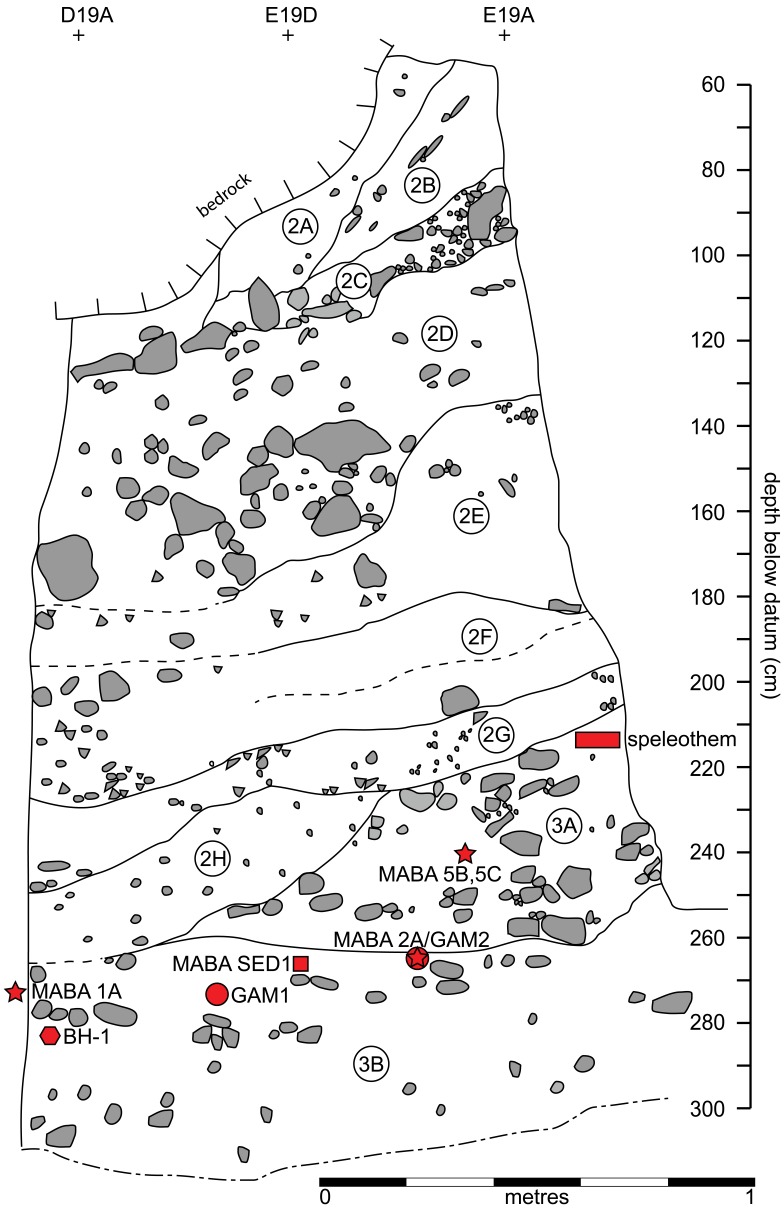
Vertical distribution of the samples on the northern profile. Northern (southwest-facing) profile as shown in Figure 1. Limestone gravel is shown schematically to represent physical arrangement of the coarse components with the fine-grained sediment matrix (white). Geological layers (GH) are shown (encircled numbers), and the locations of dated sediment, teeth and speleothem samples as a function of depth, except MABA 1A which projects outside the limit of the profile. The speleothem was found in layer 3a as shown here diagrammatically, but the dating sample was recovered from near the eastern profile as shown in Figure 1. GAM means location of gamma spectrometer measurement.

Figure 1 shows the site location and plan of the excavations with the positions of dated samples and the BH-1 mandible, while Figure 2 shows their locations projected onto the northern profile of the excavations. The BH-1 mandible was found at a depth of −281 to −285 cm. in geological layer (GH) 3b (Figure 2). Four enamel samples were dated: MABA 1A, 2A, 5B and 5C (two subsamples of tooth 5). Each were dated using two combined ESR/Uranium Series techniques: US-ESR [14] and CSUS-ESR [15], each technique employing a different uranium uptake modeling method. These samples were taken from the area surrounding the mandible, but from above it, within layers 3b, 3a/b, and 3a respectively (Figures 1 and 2, Table 1).

**Table 1:** Age Results for Dental Enamel, Sediment and Flowstone at Mala Balanica.

Sample	Rink Stal 4Flowstone	Maba 5B EN	Maba 5C EN	Maba 2A EN	Maba SED 1	Maba 1A EN
Depth (cm)	203–216	240	240	266	270	272
Geol. Horiz.	3a	3a3	3a3	3a3/3b	3b	3b
p-value EN		−0.77+/−0.09	−0.75+/−0.09	−0.16+/−0.20		
p-value DEN		−0.47+/−0.13	−0.45+/−0.11	−0.02+/−0.12		−0.43+/−0.18
CS 230^Th^/234U Age (ka)*1	c. 350–600					
CSUS ESR Age (ka)*2		610+/−110	611+/−99	553+- 49		437+/−79
US-ESR Age (ka)*3		395+59/−56	413+54/−52	482+43/−39		383+70/−63
US-ESR Age Range (ka)*4		339–454	361–467	443–525		320–453
IRSL Age (ka)*5					449+/−52	

*1 Closed system age based only on isotopic ratios.

*2 Closed system uranium series ESR age: special case based on assumptions explained in text and using isotopic ratios and ESR parameters, based on [15].

*3 US-ESR Age based on modeled p-values [14] ranging over ESR dose parameters and isotopic ratios (most widely used).

*4 Same as 3 but time range including all age uncertainty.

*5 Infrared stimulated luminescence age based on the Post IR/IR procedure and assuming a 1% fading correction as discussed in the text and Text S1.

MABA SED 1 was also obtained from above the mandible, from the same area within layer 3b. It was dated using the infrared/post infrared (IR/post IR) luminescence dating procedure [16].

Finally, our uppermost dated sample (STAL 4) is a coarsely-crystalline carbonate flowstone sample from the upper portion of layer 3a. It was dated using closed-system assumption ^230^Th/^234^U dating [17]. Sample depths, lithological units (or layers), taxa and depths below datum are given in Table S1.

Basic ESR sample preparation followed Rink et al. [18]. Table S2 reports analytical data used as input values for the software described in Grün [19] that yielded the US-ESR and CSUS-ESR results. Table S3 provides dosimetry results and dose rate results. Sample preparation for isotopic analysis of ^230^Th/^234^U ratios and other isotopic measurements were carried out at the Université du Québec à Montréal (UQAM) using a thermal ionization mass spectrometer equipped with a secondary electron multiplier (see Text S1). Table S4 provides isotopic results from UQAM. The sediment was prepared and dated at the University of Bordeaux following protocols described in Text S1. All necessary permits were obtained for the described field studies. Permission was granted by the Ministry of Culture of the Republic of Serbia (permission number: 633-00139/2010-03).

Additional details of the ESR, isotopic and infrared stimulated luminescence sample preparation and measurements are found in Text S1. Figures S1 and S2 show dose response data and curve fitting for ESR and infrared/post infrared luminescence (hereafter referred to as IRSL) measurements respectively. In-situ gamma dosimetry was independently performed for ESR dating by WJR and by NM for the IRSL dating, and is discussed in Text S1.

## Results

### Stratigraphic Context of the Dated Samples

Detailed geoarchaeological analyses of the Balanica sedimentary sequence are currently in progress. However, a preliminary field assessment of the sequence affords some insights into the depositional environments represented in the sequence at Mala Balanica. At the base of the sequence (layer 3c and below), thick (>1m), bedded fine silt and clay units are present (recorded in an auger hole taken in front of the section), which show that prior to the deposition of layers 3a and 3b the Sićevo Gorge area experienced particularly humid conditions and as a consequence water pooled in this area of the cave close to the bedrock floor.

Overlying layer 3c, the lower part of the excavated sequence (layers 3b –3a) comprises fine-grained silts and sands, with a medium, sub-angular to sub-rounded limestone gravel component. This shows a marked change in depositional environment, from the low-energy regimes represented by the fine-grained sediments of layers 3c and below, to a much more dynamic environment characterised by increasing coarse sediment deposition. Layer 3a is recorded at 200–210 cm b.d. (below datum) in the central part of the cave, whilst near the western wall it is present at 240–260 cm b.d. This suggests that a talus cone which is evidenced in upper layers 2h –2b started to form at this time towards the central area of the site, most likely as a function of climatic deterioration promoting increased cryoclastic activity.

The remains of a laterally extensive speleothem (flowstone) were recorded within the upper part of layer 3a. Speleothem fragments were also found in the central area of the cave, but not near the western wall, possibly suggesting rapid burial and preservation beneath the debris cone. However, excavations in 2010 and 2011 demonstrated that layer 3b was intact all the way to the cave wall. This confirms that following the deposition of layer 3a the interior site dynamics changed markedly.

The upper part of the Pleistocene sedimentary sequence (layers 2h –2b) is dominated by coarse, sub-angular to angular limestone gravel suspended within a matrix of reddened silts and sands. The size and shape of the gravel components are consistent with deposition under a cold climatic regime, and may broadly indicate a climatic downturn following the deposition of layers 3a –3c. Some layers (e.g. 2b) are clast-supported, containing very low quantities of fine material, most likely reflecting particularly active periods of host bedrock attrition. This is borne out by the inclination of bedding planes and imbrication of gravel clasts in layers 2e –2g, confirming deposition at the distal end of a debris cone situated further out in the central area of the cave.

## Dating Results

The age of the mandible is best constrained using the US-ESR age of MABA 2A and the sediment sample MABA SED 1, which both occur only slightly higher in the deposit than the mandible (Table 1, Figure 2). Our MABA SED 1 sample at −270 cm yields an age of 449±52 ka (range 397–501 ka), while the US-ESR age of MABA 2A (at −266 cm) is 482+43/−39 ka (range 441–525 ka). Therefore, based on the principle of superposition, and combining the results from MABA 2A and MABA SED 1, we obtain the best minimum age estimate of the underlying mandible to be 397–525 ka. Other dated samples are consistent with this age estimate. MABA 1A at −272 cm has a US-ESR age of 383+70/−63 ka (range 320–446 ka), and a tooth higher in the section at −240 cm yielded two subsample US-ESR ages of 395+59/−56 ka (range 339–454 ka) and 413+54/−52 ka (range 361–467 ka) - MABA 5B and C respectively. Finally a carbonate flowstone fragment located even higher in the deposit at −203 to −216 cm yielded ^230^Th/^234^U isotopic ratios consistent with an age range near the limit of the method in calcite speleothems of around 350–600 ka.

The age of the lowest tooth (MABA 1A; 383+70/−63 ka), located slightly above the mandible, is probably an underestimate due to the fact that the gamma spectrometric measurements used in its age determination are likely an overestimate (the tooth came from near bedrock, while the lowest position in which gamma spectrometric measurements could be made in profiles was not as close to bedrock as desired). We therefore reject the US-ESR and CSUS-ESR age estimates for this tooth from further consideration in the interpretation. However, it is still in agreement with the best age estimate on the next higher tooth (MABA 2A) and the sediment IRSL age, which we have used to constrain the minimum age of the mandible at 397–525 ka.

A final consideration for the minimum age of the BH-1 human mandible arises with respect to the results in Table 1 that were obtained using the CSUS-ESR model [15] for the burial age estimates. This model assumes that all of the uranium in the tooth was taken up instantaneously at the time indicated by the apparent closed-system ^230^Th/^234^U age of the dental tissues. This yields a true maximum possible burial age because it accounts for a possible delayed uptake of U not accounted for in the parametric functions used in the US-ESR ages that provide for continuous smooth uptake of uranium. In effect, the CSUS-ESR model provides a test of the robustness of the US-ESR ages (Table 1). If CSUS-ESR model ages are generally in agreement with the US-ESR ages, there is good reason to believe they are the best age estimates in a sequence. The best agreement is found for MABA 2A, for which the CSUS-ESR age is 553±49 and the US-ESR age is 482+43/−39 ka. For the MABA 5 subsamples, the CSUS-ESR ages are considerably older than their counterpart US-ESR ages. To summarize the US-ESR dating results, the age of MABA 2A at 443–525 ka is the best minimum age estimate among the three teeth studied here.

We have also considered the possibility that the CSUS-ESR age of MABA 2A constrains the maximum possible age above the BH-1 human mandible to be 553±49 ka. This produces the maximum value of 602 ka, suggesting that the mandible could be older than this age due to its stratigraphically lower position. However, because the CSUS-ESR model involves an extreme assumption that all uranium was taken up at the time of its apparent U-series age (Closed System ^230^Th/^234^U Age of Table S4), we do not favor this interpretation.

Considering the age of the MABA SED 1 sediment sample (449±52 ka) does not fully resolve the question of the mandible’s minimum possible age, even though this result is not affected by the uncertainties associated with the uranium uptake modeling. In fact, the sediment age was obtained assuming a fading rate of the measured IRSL signal of 1%, but values up to 2% have been estimated [16]. Using this last value would yield an age of 521±61 ka, and would be considered to be a maximum age for sediment deposition above the mandible. This indicates that BH-1 could be older than 582 ka based on adding the uncertainty of +61 ka to the value of 521ka.

Combining the results for the US-ESR age of MABA 2A and the IRSL age for SED 1, we obtain a minimum age estimate for the BH-1 mandible of 397–582 ka. This incorporates all of the uncertainty in the two IRSL sediment estimates (1% and 2% fading), and because the fading value remains unknown (likely between 1 and 2%), the most conservative approach would be to include the time interval covered by these two possibilities, i.e. 397–582 ka. If we were to exclude the possibility of a fading correction of 2%, this yields an age range of 397 to 525 ka. The 397–582 ka range encompasses all of the uncertainty in the tooth age alone, whose age range is 443–525 ka (US-ESR model). Though the mandible could be as old as 582 ka, we favor an interpretation that the minimum age of the mandible lies between 397 and 525 ka. This interpretation is strongly supported by the US-ESR ages of 395+59/−56 and 413+54/−52 for the overlying samples from tooth MABA 5 (MABA 5B and 5C), that was found about 25 cm higher in the deposit than MABA 2A and MABA SED1. We suggest that others should cite the age of the BH-1 mandible as “BH1 has a minimum age that lies between 397 and 525 ka.”

This result of >397–525 ka is significantly older but still consistent with a previous attempt [12] to determine the age of the BH-1 human mandible based on non-destructive gamma spectrometric analysis of the ^238^U, ^234^U, and ^230^Th concentrations in the mandible itself [12], which resulted in a minimum age of 113+72/−43 ka.

## Discussion

The minimum age range of 397–525 ka places BH-1 mandible firmly among the oldest hominin fossils in Europe. The older estimate overlaps with Sima de los Huesos (600±60) [20] and is only slightly younger than Mauer (609±40) [21], while the younger minimum age limit of 397 ka overlaps with Arago (435±85) [22] and Visogliano (350–500) [23], and is somewhat older than Ceprano (353±4) [24]. BH-1 is the easternmost hominin specimen in Europe securely dated to the Middle Pleistocene. Petralona 1 and Apidima 2, the only other Middle Pleistocene specimens from this area, are notably younger: Petralona 1 is dated between 150 ka and 250/350 ka [25], and Apidima between 105 to 400 ka but more likely towards the upper limit of the date [26]. With the exception of the Visogliano mandible, which is identified as *H. erectus* [27], all of the BH-1 penecontemporary specimens are currently identified as *H.heidelbergensis,* often considered a chronospecies of Neandertal in the European context [5]. Recent advances in radiometric dating of key European Middle Pleistocene specimens, Sima de los Huesos [20], Mauer [21], Arago [22] and Ceprano [24], and detailed publication of the Sima de los Huesos material [28] challenge the notion of gradual progression towards classical Neandertal morphology [5]. Namely, the Sima de los Huesos assemblage shows more pronounced derived Neandertal morphology than the contemporaneous, but more easterly Mauer, or the later Arago or Ceprano specimens, all of which show fewer Neandertal traits. To explain this phenomenon, Dennell et al. [9] examined Middle Pleistocene variability in Europe in the light of geographically and chronologically defined p-demes, and proposed a population model that is based on demographic “sources” and “sinks.” The model proposes a small number of core “sources” in the south of the continent that re-populated more northerly areas during interglacials, with northern groups representing demographic “sinks.” Relevant for understanding the dynamics of the Balkan Peninsula is the inclusion of Southwest Asia as one of the sources of re-population. With western source populations as bearers of derived Neandertal morphology, attenuation of Neandertal traits in the more easterly or later populations was explained by admixture with a group from outside of the isolated glacial refugee, i.e. a population from Southwest Asia. Under this model, we would expect that Southeast Europe – the Balkan Peninsula – would have remained in contact with Southwest Asia during glacial episodes, or at minimum served as a transit route [9]. This places emphasis on the current fossil record of Southeast Europe that, while comparatively scant, becomes critical for understanding continent-wide processes. While isolation represented the major mechanism of evolutionary change in the West of the continent [2], causing bottleneck and fixation of derived traits, the Balkan Peninsula did not experience the effects of isolation. Accordingly, the population that inhabited the Balkan Peninsula and maintained contact with Southwest Asia throughout glaciations could have retained a number of primitive (i.e., non-Neandertal) traits, without precluding morphological changes associated with encephalization and tooth reduction observed in Middle Pleistocene populations on all three continents.

On the basis of preserved morphology, BH-1 differs significantly from Middle Pleistocene European hominins generally grouped under *H. heidelbergensis* [12]. It exhibits primitive features such as a prominent *planum alveolare*, thick mandibular corpus, wide exomolar sulcus, flat rather than concave sublingual fossa, and poorly defined relief of the submandibular fossa. There is a complete lack of derived Neandertal features: the mental foramen is below the P_4_ alveolus, equidistant from the alveolar and the basal margins, and there is no retromolar space. Dental traits are equally plesiomorphic: mesotaurdontic roots, two mesial and two distal diverticles on the M_1,_ “Y” fissure pattern, five main cusps, and a well-developed “cusp 7.” There is a clear lack of mid-trigonid crest on all three molars, generally considered as a diagnostic feature in Neandertals [29]. Given the size of the mandibular body, the dentition is relatively small, and fits well with Middle Pleistocene European specimens.

Several specimens in close proximity show similar combination of plesiomorphic *erectus*-like and synapomorphic (Middle Pleistocene trend) morphologies. Similarly to Balanica, the mandible from Visogliano – the closest specimen both temporally and geographically – demonstrates plesiomorphic traits and a complete lack of derived Neandertal morphology [27], while the associated maxillary dentition is considered remarkably similar to *H.erectus* from Zhoukoudian Lower Cave [30]. The Ceprano cranium, originally considered to be much older [31], shows a combination of primitive *H. erectus/ergaster* features in midsaggital profile – such as fronto-parietal flattening and the development of supraorbital and nuchal structures – combined with synapomorphic frontal bone traits such as widening of the frontal squama [32]. Currently considered to be *H. heidelbergensis* [33], the Ceprano cranium fits well with these specimens as it shows either plesiomorphic or synapomorphic features but no derived Neandertal traits. This grouping could tentatively include the Kocabaş specimens from Anatolia [33]. While the calvarium was not directly dated, the travertine layer in the zone of its origin was dated to 1.11±0.11 Ma (Lower Pleistocene) by ESR [35] and to 510±50 ka and 330±30 ka (Middle Pleistocene) by thermoluminescence of the calcite in the travertine (Özkul et al 2004a cited in [34]). Though these techniques remain experimental on these particular materials, there is apparently some other faunal evidence that supports the time attribution to Middle-Lower Pleistocene [35]. Although only a limited number of measurements could be made, the specimen is both metrically and morphologically consistent with Asian *H. erectus* [34]. The crania from Petralona and Apidima, with their strong Neandertal affinities, especially in the facial region, coupled with the presence of Krapina in the adjoining Western Balkans at 130 ka [36], could bear evidence of successful eastward spread of Neandertals in the later part of the Middle Pleistocene.

### Conclusions

The newly obtained minimum age of 397–525 ka for the BH-1 hominin fossil from Balanica Cave complex, Serbia, makes this specimen at least as old as the central third (from about 350 to 560 ka) of the Middle Pleistocene (130 to 780 ka). It is broadly contemporaneous with other radiometrically dated specimens such as Sima de los Huesos, Mauer, Arago, Ceprano and Visogliano. BH-1 represents one of an increasing number of specimens from the southeast of the continent demonstrating plesiomorphic traits coupled with synapomorphic traits common to Middle Pleistocene hominins (such as encephalization and dental reduction). With a complete lack of derived Neandertal traits, these specimens are distinct from the more westerly penecontemporary hominins. Although the sample size is small, and consists of unassociated crania and mandibles, this pattern is consistent with a lack of isolation during glaciations that resulted in different morphological outcomes from those at the west of the continent. In that context, the Balkan Peninsula could be part of the geographic spread of a Southwest Asian “source” population [9] for the purported successive repopulation of Europe in the Middle Pleistocene.

## Supporting Information

Figure S1 **Electron spin resonance signal intensity (arbitrary units) as a function of added dose, fitted with a single saturating exponential function.**
(TIF)

Figure S2 **Normalized Infrared Luminescence/Post Infrared Luminescence Ratios a function of regeneration dose.**
(TIF)

Table S1 **Dating Sample and Gamma Spectrometer** ***in-situ*** **Dose Rate Locations (GAM) for Mala Balanica, Serbia.**
(DOC)

Table S2 **US-ESR and CSUS-ESR Age Calculation Input Data for Mala Balanica, Serbia.**
(DOC)

Table S3 **In-situ, US-ESR and Sediment Dosimetry Results for Mala Balanica, Serbia.**
(DOC)

Table S4 **Isotopic Data for Enamel and Dentine and Calcite Speleothem Mala Balanica, Serbia.**
(DOC)

Text S1 [**37**]–[**45**] **.**
(DOC)
