# Correction: New Radiometric Ages for the BH-1 Hominin from Balanica (Serbia): Implications for Understanding the Role of the Balkans in Middle Pleistocene Human Evolution

**DOI:** 10.1371/annotation/c940fe44-4832-4234-9e05-870f09268be1

**Published:** 2013-04-09

**Authors:** William J. Rink, Norbert Mercier, Dušan Mihailović, Mike W. Morley, Jeroen W. Thompson, Mirjana Roksandic

There was an error in the Funding Statement. The correct funding is:

Natural Sciences and Engineering Council of Canada to WJR and MR (grant numbers not disclosed). DM received financial support from the Serbian Ministry of Culture and Ministry of Science, Project 177023. The funders had no role in study design, data collection and analysis, decision to publish, or preparation of the manuscript.

A word was misspelled in the Abstract. "**Vasogliano**" should be "**Visogliano**"

There was an error in the Supporting Information. In Table S1, in the last column of the row "BH-1 Mandible", the value "*-218*" should be "*-281*"

