# Supplementary figures and images for: New Radiometric Ages for the BH-1 Hominin from Balanica (Serbia): Implications for Understanding the Role of the Balkans in Middle Pleistocene Human Evolution
Source: PLoS One. 2013 Feb 6;8(2):e54608. doi: 10.1371/journal.pone.0054608 (PMC3566111; doi:10.1371/journal.pone.0054608)

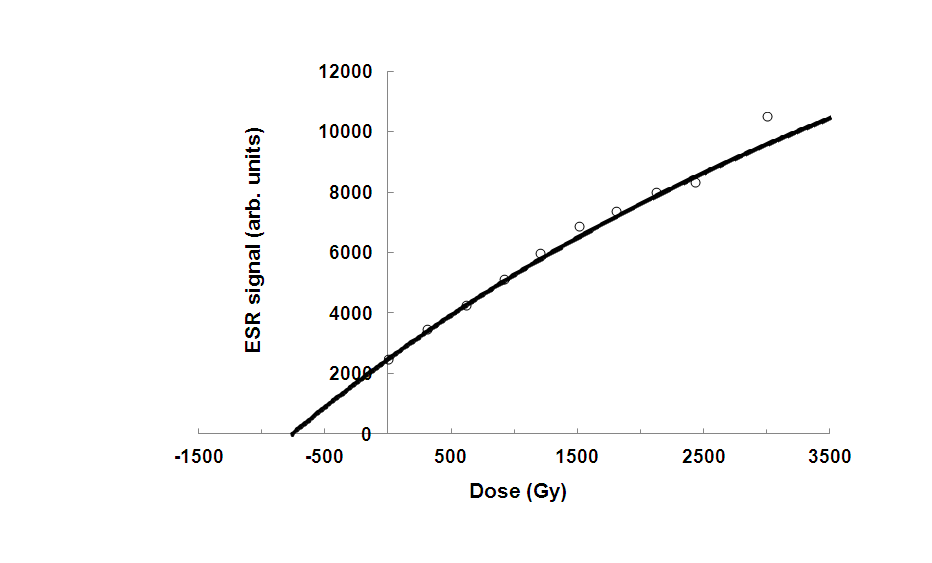

Supplement: Figure S1 — Electron spin resonance signal intensity (arbitrary units) as a function of added dose, fitted with a single saturating exponential function. (TIF) [file pone.0054608.s001.tif]

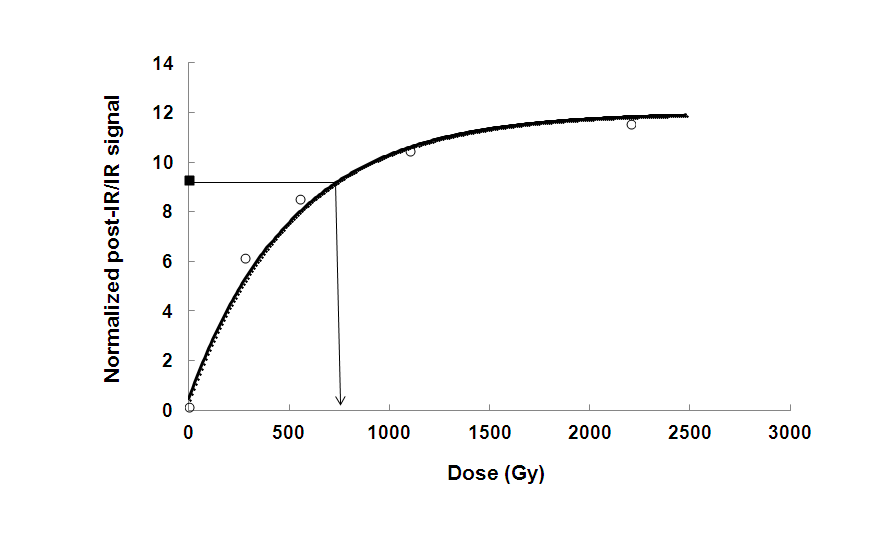

Supplement: Figure S2 — Normalized Infrared Luminescence/Post Infrared Luminescence Ratios a function of regeneration dose. (TIF) [file pone.0054608.s002.tif]
