# Supplementary material for: New Radiometric Ages for the BH-1 Hominin from Balanica (Serbia): Implications for Understanding the Role of the Balkans in Middle Pleistocene Human Evolution
Source: PLoS One. 2013 Feb 6;8(2):e54608. doi: 10.1371/journal.pone.0054608 (PMC3566111; doi:10.1371/journal.pone.0054608)
Supplement: Table S1 — Dating Sample and Gamma Spectrometer in-situ Dose Rate Locations (GAM) for Mala Balanica, Serbia. (DOC) [file pone.0054608.s003.doc]

Table S1. Dating Sample and Gamma Spectrometer *in-situ* Dose Rate Locations (GAM) for Mala Balanica, Serbia

| Sample | IS# | GH | Evidence  List No. | Square | Type or Taxon | x  (cm) | y  (cm) | z (Depth ) (cm) |
| --- | --- | --- | --- | --- | --- | --- | --- | --- |
| Rink Stal 4 | n/a | 3a | n/a | F18c | Stalagmitic Floor | 56 | 54 | -203 to -216 |
| Maba 5B | 10 | 3a3 | 196e | E19b | Ursus  arctos | 7 | 83 | -240 |
| Maba 5C | 10 | 3a3 | 196e | E19b | Ursos  arctos | 7 | 83 | -240 |
| Maba GAM 5 | n/a | 3a | n/a | F17d | n/a | 35 | 6 | -239 |
| Maba 2A | 20 | 3a3/3b | 233e | E18b | Capra  Ibex | 20 | 52 | -266 |
| Maba GAM 2 | n/a | 3a/3b | n/a | E20c | n/a | 63 | 95 | -261 |
| Maba SED 1 | n/a | 3b | n/a | E19a | Sediment | 92 | 95 | -270 |
| Maba 1A | 9 | 3b | 239e | C18b | Ibex | 5 | 51 | -272 |
| Maba GAM 1 | n/a | 3b | n/a | D20b | n/a | 9 | 95 | -261 |
| BH-1 Mandible | n/a | 3b | n/a | D18 | Homo | 15-45 | 15-55 | -218 to -285 |

Footnotes:

IS# = original specimen number

GH = Geological horizon

n/a = not applicable
