# Supplementary material for: New Radiometric Ages for the BH-1 Hominin from Balanica (Serbia): Implications for Understanding the Role of the Balkans in Middle Pleistocene Human Evolution
Source: PLoS One. 2013 Feb 6;8(2):e54608. doi: 10.1371/journal.pone.0054608 (PMC3566111; doi:10.1371/journal.pone.0054608)
Supplement: Table S2 — US-ESR and CSUS-ESR Age Calculation Input Data for Mala Balanica, Serbia. (DOC) [file pone.0054608.s004.doc]

Table S2. US-ESR and CSUS-ESR Age Calculation Input Data for Mala Balanica, Serbia

| **Tooth Name** | **Maba 5B** | **Maba 5C** | **Maba 2A** | **Maba 1A** |
| --- | --- | --- | --- | --- |
| **De (Gy)** | 762.22 +/- 77.44 | 708.36 +/- 63.64 | 465.78 +/- 21.86 | 493.30 +/- 60.18 |
| **Enamel ^238^U (ppm)** | 1.3698 +/- 0.0064 | 1.3818 +/- 0.0061 | 0.2845 +/- 0.0012 | < 0.02 (0 for calculation) |
| **Enamel ^234^U/^238^U** | 1.1241 +/- 0.0173 | 1.0933 +/- 0.0148 | 1.2794 +/- 0.0101 | n/a |
| **Enamel ^230^Th/^234^U** | 0.9198 +/- 0.0144 | 0.9161 +/- 0.0170 | 0.8222 +/- 0.0337 | n/a |
| **Dentine ^238^U (ppm)** | 51.1556 +/- 0.2439 | 49.4324 +/- 0.2184 | 17.7073 +/- 0.0676 | 23.0650 +/- 0.1790 |
| **Dentine ^234^U/^238^U** | 1.0433 +/- 0.0087 | 1.0414 +/- 0.0071 | 1.1623 +/- 0.0075 | 1.1026 +/- 0.0212 |
| **Dentine ^230Th^/^234^U** | 0.8283 +/- 0.0069 | 0.8332 +/- 0.0060 | 0.7907 +/- 0.0074 | 0.8181 +/- 0.0157 |
| **Enamel Thick.**  **micrometers)** | 656 +/- 114 | 825 +/- 67 | 828 +/- 106 | 504 +/- 184 |
| **Sed. Side Removed**  **(micrometers)** | 0 | 32 +/- 6 | 35 +/- 7 | 60 +/- 12 |
| **Den. Side Removed**  **(micrometers)** | 52 +/- 10 | 27 +/- 5 | 57 +/- 12 | 80 +/- 16 |
| **Sediment ^238^U (ppm)** | 6.12 +/- 0.1 | 6.12 +/- 0.1 | 4.88 +/- 0.1 | 8.83 +/- 0.1 |
| **Sediment ^232^Th (ppm)** | 2.52 +/- 0.17 | 2.52 +/- 0.17 | 5.61 +/- 0.37 | 6.80 +/- 0.44 |
| **Sediment ^40^K (ppm)** | 0.47 +/- 0.01 | 0.47 +/- 0.01 | 0.97 +/- 0.03 | 1.13 +/- 0.03 |
| **Sediment Moisture**  **(wt. %)** | 8.0 +/- 5 | 8.0 +/- 5 | 14.0 +/- 5 | 12.0 +/- 5 |
| **Gamma Dose Rate**  **(microGrays/year)** | 422 +/- 22 | 433 +/- 43 | 435 +/- 44 | 416 +/- 42 |
| **Burial Thickness (m)** | 6 +/- 1 | 6 +/- 1 | 6 +/- 1 | 6 +/- 1 |
