# Supplementary material for: New Radiometric Ages for the BH-1 Hominin from Balanica (Serbia): Implications for Understanding the Role of the Balkans in Middle Pleistocene Human Evolution
Source: PLoS One. 2013 Feb 6;8(2):e54608. doi: 10.1371/journal.pone.0054608 (PMC3566111; doi:10.1371/journal.pone.0054608)
Supplement: Table S3 — In-situ, US-ESR and Sediment Dosimetry Results for Mala Balanica, Serbia. (DOC) [file pone.0054608.s005.doc]

Table S3. *In-Situ*, US-ESR, and Sediment Dosimetry Results for Mala Balanica, Serbia

| **Tooth**  **Sample Name** | **In-situ**  **Gamma Dose Rate**  **(1 x 10^-6^Gy/ a)** | **Sediment**  **Beta Dose Rate**  **(1 x 10^-6^Gy/a)** | **Cosmic Dose Rate**  **(1 x 10^-6^Gy/a)** | **Enamel Alpha Plus Beta Dose Rate**  **(1 x 10^-6^Gy/a)** | **Dentine Beta Dose Rate**  **(1 x 10^-6^Gy/a)** | **Internal Beta Dose Rate to**  **Sediment Grains**  **(1 x 10^-6^Gy/a)** | **External Alpha Dose Rate to Sediment Grains**  **(1 x 10^-6^Gy/a)** | **Total Dose Rate**  **(1 x 10^-6^Gy/a)** |
| --- | --- | --- | --- | --- | --- | --- | --- | --- |
| **Maba 4A** | 754 +/- 75 | 294 +/- 58 | 97 +/- 6 | 59 +/- 10 | 271 +/- 64 |  |  | 1476 +/- 95 |
| **Maba 5B** | 433 +/- 43 | 206 +/- 27 | 97 +/- 6 | 450 +/- 86 | 741 +/- 139 |  |  | 1927 +/- 167 |
| **Maba 5C** | 433 +/- 43 | 189 +/- 16 | 97 +/- 6 | 441 +/- 83 | 554 +/- 73 |  |  | 1715 +/- 120 |
| **Maba 2A** | 435 +/- 44 | 195 +/- 24 | 97 +/- 6 | 68 +/- 17 | 169 +/- 26 |  |  | 965 +/- 59 |
| **Maba SED 1** | 428 +/- 53 | 1266 +/- 40 | 97 +/- 6 | n/a | n/a | 111 +/- 14 | 121 +/- 19 | 2022 +/- 71 |
| **Maba 1A** | 416 +/- 42 | 413 +/- 86 | 97 +/- 6 | 0 | 359 +/- 99 |  |  | 1285 +/- 38 |

Table Footnotes: N/a = not applicable
