# Supplementary material for: New Radiometric Ages for the BH-1 Hominin from Balanica (Serbia): Implications for Understanding the Role of the Balkans in Middle Pleistocene Human Evolution
Source: PLoS One. 2013 Feb 6;8(2):e54608. doi: 10.1371/journal.pone.0054608 (PMC3566111; doi:10.1371/journal.pone.0054608)
Supplement: Table S4 — Isotopic Data for Enamel and Dentine and Calcite Speleothem Mala Balanica, Serbia. (DOC) [file pone.0054608.s006.doc]

Table S4. Isotopic Data for Enamel, Dentine and Calcite Speleothem (Flowstone) Mala Balanica, Serbia

| **Sample Name** | **Sample Type** | **^238^U (ppm)** | **^230^Th/^232^Th**  **Activity Ratio** | **^234^U/^238^U**  **Activity Ratio** | **^230^Th/^234^U**  **Activity Ratio** | **Closed System ^230^Th/^234^U Age (ka)** |
| --- | --- | --- | --- | --- | --- | --- |
|  |  |  |  |  |  |  |
| Maba 5B | Enamel | 1.3698 +/- 0.0064 | 581.626 +/- 3.747 | 1.1241 +/- 0.0173 | 0.9198 +/- 0.0144 | 243.829 + 22.693 / - 19.291 |
|  | Dentine | 51.1556 +/- 0.2439 | 77608.447 +/- 609.802 | 1.0433 +/- 0.0087 | 0.8283 +/- 0.0069 | 187.633 + 6.617 / - 6.245 |
|  |  |  |  |  |  |  |
| Maba 5C | Enamel | 1.3818 +/- 0.0061 | 1483.935 +/- 21.382 | 1.0933 +/- 0.0148 | 0.9161 +/- 0.0170 | 246.137 + 23.962 / - 20.428 |
|  | Dentine | 49.4324 +/- 0.2184 | 39624.396 +/- 282.368 | 1.0414 +/- 0.0071 | 0.8332 +/- 0.0060 | 190.771 + 5.716 / - 5.446 |
|  |  |  |  |  |  |  |
| Maba 2A | Enamel | 0.2845 +/- 0.0012 | 140.128 +/- 5.751 | 1.2794 +/- 0.0101 | 0.8222 +/- 0.0337 | 168.704 + 16.633 / - 14.709 |
|  | Dentine | 17.7073 +/- 0.0676 | 4623.682 +/- 45.715 | 1.1623 +/- 0.0075 | 0.7907 +/- 0.0074 | 160.206 + 4.233 / - 4.096 |
|  |  |  |  |  |  |  |
| Maba 1A | Enamel | 0 | n/a | n/a | n/a | n/a |
|  | Dentine | 23.0650 +/- 0.1790 | 3498.896 +/- 35.006 | 1.1026 +/- 0.0212 | 0.8181 +/- 0.0157 | 176.878 + 13.509 / - 12.041 |
|  |  |  |  |  |  |  |
| Rink Stal 4 | Flowstone | 0.5290 +/- 0.0020 | 149.424 +/- 1.794 | 0.9878 +/- 0.0066 | 1.0131 +/- 0.0115 | > 350 to 600 |

Table Footnotes: n/a = not applicable
