# Supplementary material for: New Radiometric Ages for the BH-1 Hominin from Balanica (Serbia): Implications for Understanding the Role of the Balkans in Middle Pleistocene Human Evolution
Source: PLoS One. 2013 Feb 6;8(2):e54608. doi: 10.1371/journal.pone.0054608 (PMC3566111; doi:10.1371/journal.pone.0054608)
Supplement: Text S1 — [37]–[45] . (DOC) [file pone.0054608.s007.doc]

Text S1

**1. Electron Spin Resonance (ESR) Sample Preparation and Measurements on Dental Samples, and** *in-situ* **dosimetric measurements related to the ESR dating work.**

Sample preparation followed procedures outlined in [18]. After crushing, the different portions of each powdered enamel sample were irradiated using a ^60^Co source at the McMaster University Nuclear Reactor. The added doses given (in Gray) were: 302.5, 612.6, 918.0, 1201.0, 1511.0, 1804.0, 2116.0, 2429.0, 2999.0, and then stored for 2 weeks.

The measurements were made using a JEOL JES-FE100 x-band spectrometer, operating near 9.4 x 10^9^ Hz microwave frequency. The g=2.0018 signal was measured using the following experimental conditions: microwave power = 5.0 milliwatt, field scan width = 5 x 10^-3^ Tesla, modulation frequency 100,000 Hz, modulation amplitude 0.5 x 10^-3^ Tesla, time constant = 0.3 seconds, and scan rate = 5 x 10^-3^ Tesla/minute. The signal intensity was determined as the y-axis peak to peak intensity of the resonance at g=2.0018. The equivalent dose for each sample was determined by fitting a single-saturating exponential function (weighting = 1/intensity^2^), and the equivalent dose error was determined using the method of Brumby [37]. The dose response data for MABA 5B enamel are shown in Figure S1. The equivalent dose from this sample is the x-axis intercept value of 762.22 Gray.

The sediments attached to the enamel were analysed for ^238^U, ^232^Th and ^40^K using delayed neutron counting (U) and instrumental neutron activation analysis (Th and K). Radioactive equilibria in the decay chains of ^238^U and ^232^Th were assumed. The enamels and dentines were initially analysed for ^238^U by delayed neutron counting, and later by thermal ionization mass spectrometric analysis (except for Maba 1A whose initial analysis by delayed neutron counting indicated essentially zero uranium concentration). See section 2 of this text for details of the mass spectrometric analysis. The moisture content of the sediment samples was directly measured on sediment (Table S2), whereas the moisture content in the dentine was assumed.

Table S1 and Figure 2 show the relationship between the sample locations and the in-situ gamma dose rate measurements. The GAM 1, 2 and 5 measurements were made using a Harwell gamma spectrometer converted to a scintillometer, calibrated using a triple point calibration obtained in the concrete blocks at the Oxford Research Laboratory for Archaeology and the History of Art. A count from a monazite sand sample standard is used on-site to insure that the internal self-calibration during startup of the instrument is behaving normally, and has not been compromised by any damage during travel to the site. The scintillometer employs a 2.0 inch sodium iodide crystal, and a correction is made to remove the counts from cosmic ray contributions. The vertical measurement positions in existing profiles were chosen near the sample elevation and laterally along a nearby profile to best approximate the lithology at the elevations where the samples were collected during routine excavations. These excavations had occurred 1-3 years before the dating program began in 2010. The horizontal sample to gamma spectrometer position distances ranged from 1.4 to 2.1 meters.

All of the results of the sample preparation measur**e**ments and ESR measurement data, along with the results of the isotopic measur**e**ments, are listed in Table S2. The input data in Table S2 was used to calculate both the US-ESR Ages [14] and the CSUS-ESR Model Ages [15] in Table 1. No results are presented for MABA 1 enamel with respect to p-value (Table 1), nor for isotopic ratios and closed system ^230^Th/^234^U ages (Tables S2 and S4) because it was found to have zero uranium concentration (<0.02 ppm by delayed neutron counting (Table S2) is considered zero by the analytical facility because it is below detection limit). This precluded its further analysis by isotopic methods because it would in no case contribute uranium dose in any uptake model scenario. Table S3 provides all of the radiation dosimetry results for the teeth and the sediment sample (see section 3 below).

**2. Isotopic Measurements and Sample Preparation on Dentine and Enamel Samples**

Dentine and enamel samples of nominal weights between 0.1 to 0.2 g were weighed with high accuracy into Teflon beakers and spiked with a mixed tracer of ^233^U-^236^U-^229^Th. The enamel and dentine samples were acid digested with *aqua regia,* a mixture of concentrated HNO_3_ and HCl 6N, evaporated to dryness then re-dissolved with HCl 6N. For the CaCO_3_ sample, a few grams of sample was dissolved in 7N HNO_3_ in order to concentrate the U and Th from the bulk solution, and an Fe(OH)_3_ precipitate was created by adding a solution of ammonium hydroxide until a pH between 7 and 9 was obtained. The precipitate was recovered by centrifugation and then dissolved in 6N HCl followed by the same column chemistry as the dentine and enamel samples.

The U-Th separation was conducted using an AG1X8 anionic resin bed. The Th and U-Fe fractions were retrieved by elution with 6 N HCl and H_2_O, respectively. The purification of the U fraction was done using 0.2 ml U-Teva (Eichrom® Industries) resin volume. The U-Fe separation was performed by elution using a 3N HNO_3_ (Fe fraction) and an 0.002N HNO_3_ (U fraction). The purification of Th was performed by elution using a 2 ml AG1X8 resin in 7 N HNO_3_ and elution with 6N HCl. A final purification of Th was carried out on a 0.2 ml AG1X8 resin in 7N HNO_3_ and Th was eluted with 6N HCL.

The U and Th fractions were deposited on a Re filament between two layers of graphite and measured in peak jumping mode using a Triton™ Plus thermal-ionization mass spectrometer (TIMS) equipped with a secondary electron multiplier (SEM). Mass fractionation for U was corrected using a double spike of ^236^U/^233^U with an isotopic ratio of 1.1322, while mass fractionation for Th was considered negligible with respect to analytical error. The overall analytical reproducibility, as estimated from replicate measurements of standards, is usually better than 0.5% for U concentration and ^234^U/^238^U ratios, and ranges from 0.5% to 1% for ^230^Th/^234^U ratios (2s error range).

The results of the isotopic data are summarized in Table S4, along with the apparent U-series age calculated on the assumption that all of the uranium present in the sample was absorbed as soon as the sample was buried (Closed System Model). The ^230^Th/^232^Th activity ratios are all high, indicated very little contamination by common thorium during burial.

**3. Infrared Stimulated Luminescence Sample Preparation and Measurements on Sediment**

The bulk sediment (MABA SED 1) had a measured moisture content of 19.6% on the day that it was collected, but this varied strongly from the moisture contents collected in the ESR study, which ranged from 8 to 14%. A moisture content of 13% was used as an estimate for the moisture content over the entire burial history in the luminescence dating calculations. The potassium concentration in the feldspars was measured with x-ray dispersive scanning electron microscopy. The feldspar internal potassium concentration was found to be 8 +/- 1%. The bulk sediment radionuclides concentrations were measured using a high-purity germanium detector. The results showed U = 4.13 +/- 0.32 ppm, Th = 5.52 +/- 0.08 ppm and K = 1.002 +/- 0.022 wt %. The alpha and beta dose rates for the IRSL age calculation were obtained using the conversion factors of Guérin et al. [43]

The gamma dose rate for MABA SED 1 was determined by in-situ gamma scintillometry in nearby holes GAM 1 and GAM 2, but using the University of Bordeaux instrument, rather than the McMaster Instrument. After correction to the same moisture content of 13% measured when the holes were first made and measured using the McMaster scintillometer, excellent agreement was found in the comparison (Table S3), with GAM 1 = 416 +/- 42, GAM 2 = 435 +/- 44, and the mean value from GAM 1 and 2 (Bordeux scintillometer) yielding 428 +/- 53 (units = 1 x 10^-6^Gy/a).

The IRSL measurements were carried out on 40-60 μm polymineral grains extracted from the bulk sediment by wet sieving. This granulometric fraction was then treated with HCl and H2O2, for removing carbonates and organic matter, respectively, and the remaining grains were deposited onto 5mm diameter stainless steel discs with silicone oil. The luminescence signal emitted by feldspars was measured with a TL/OSL DA15 Risoe reader equipped with 7/59 and BG39 filters. An IR/post IR procedure [16] using a stimulation temperature of 225°C was chosen [17]. The equivalent dose (D_E_) has been determined with a single-aliquot regenerative dose (SAR) protocol of 7 cycles in which the regenerative doses were: 275, 550, 1100, 2200, 0 and 275 Gray (Gy), test dose: 24 Gy. At the end of each cycle, an IR optical bleaching at 290°C for 40 sec aimed at removing residual charges was applied. Eleven aliquots have been measured and provided individual equivalent dose values ranging from 714 to 928 Gy (mean value : 832 ± 65 Gy), i.e. a standard deviation of about 8%, and corrected for a residual dose of 4 Gy determined on aliquots previously bleached with a solar simulator. The dose response data are shown in Figure S2.

The IR _225°C_/post-IR signal is also known to be slightly affected by a fading rate of about 1% [18]. Applying the DRC correction of Lamothe et al. [19], the corrected mean D_E_ is 907 ± 71 Gy (1% fading). We did not attempt to measure the fading rate because its value is of the same order as the reproducibility of the measurments, and results are often equivocal. Thus we have calculated the age using two different fading rates: 1% and 2%, and reflected the results obtained in the text of the manuscript. Uncertainty in the age calculations are based on Appendix B of (Aitken, 1985), and these are derived using the recommendations for combining individual error terms indicated therein.
